# THE DEVELOPMENT OF RELOAD-C, A WEB-BASED PLATFORM TO REDUCE LONELINESS IN CAREGIVERS OF PERSONS WITH ADRD

**DOI:** 10.1093/geroni/igad104.1392

**Published:** 2023-12-21

**Authors:** Allison Marziliano, Allison Applebaum, Edith Burns, Marzena Gieniusz, Jessica Mongelli, Priya Patel, Huma Babar, Michael Diefenbach

**Affiliations:** Northwell Health, Manhasset, New York, United States; Memorial Sloan Kettering Cancer Center, New York City, New York, United States; Zucker School of Medicine at Hofstra/Northwell, Manhasset, New York, United States; Hofstra-Northwell, New Hyde Park, New York, United States; Northwell Health, Manhasset, New York, United States; Northwell Health, Manhasset, New York, United States; Northwell Health, Manhasset, New York, United States; Northwell Health, Manhasset, New York, United States

## Abstract

There is a need for effective evidence-based interventions to reduce loneliness in caregivers of persons with Alzheimer’s Disease and Related Dementias (ADRD). Interventions to reduce loneliness may be strengthened by incorporating concepts from Meaning-Centered Psychotherapy (MCP), effective in increasing meaning in life in patients with advanced cancer and their caregivers. Our central hypothesis is that concepts from MCP can be adapted for caregivers of persons with ADRD to reduce their loneliness through increasing their sense of meaning and purpose in life. Our purpose is to describe the development of RELOAD-C (REducing LOneliness in Alzeheimer’s Disease-Caregivers), a web-based platform that delivers MCP concepts, adapted for ADRD caregivers, via 6 brief videos, 7 virtual peer group meetings, and written content. The development of RELOAD-C occurred in 3 stages: 1) Preparatory Work/Adaptation of scripts from 6 existing MCP videos. 2) Stakeholder Involvement. N=15 AD/ADRD caregivers underwent individual interviews to provide expert feedback on the scripts. 3) Development of RELOAD-C. In collaboration with web specialists, RELOAD-C is created by integrating adapted MCP videos, links to virtual peer groups to discuss MCP concepts, and written MCP content. Input from stakeholders indicated: 1) preference for terminology (e.g., “caregiver” over “care partner”); 2) removing MCP content that urges caregivers to engage in complex conversations with the care recipient; 3) providing an explanation of ambiguous concepts (e.g., “unfinished business”); and 4) re-ordering the delivery of the MCP concepts. RELOAD-C is the product of multiple rounds of review by a multidisciplinary team and ADRD caregivers as expert stakeholders.

